# Circ-ZNF609 regulates G1-S progression in rhabdomyosarcoma

**DOI:** 10.1038/s41388-019-0699-4

**Published:** 2019-01-22

**Authors:** Francesca Rossi, Ivano Legnini, Francesca Megiorni, Alessio Colantoni, Tiziana Santini, Mariangela Morlando, Gaia Di Timoteo, Dario Dattilo, Carlo Dominici, Irene Bozzoni

**Affiliations:** 1 Department of Biology and Biotechnology Charles Darwin, Sapienza University of Rome, Rome, Italy; 2 Department of Pediatrics, Sapienza University of Rome, Rome, Italy; 3 Center for Life Nano Science@Sapienza, Istituto Italiano di Tecnologia, Rome, Italy

**Keywords:** Non-coding RNAs, Cancer, RNAi

## Abstract

Circular RNAs (circRNAs) represent a class of covalently closed RNAs, derived from non-canonical splicing events, which are expressed in all eukaryotes and often conserved among different species. We previously showed that the circRNA originating from the ZNF609 locus (circ-ZNF609) acts as a crucial regulator of human primary myoblast growth: indeed, the downregulation of the circRNA, and not of its linear counterpart, strongly reduced the proliferation rate of in vitro cultured myoblasts. To deepen our knowledge about circ-ZNF609 role in cell cycle regulation, we studied its expression and function in rhabdomyosarcoma (RMS), a pediatric skeletal muscle malignancy. We found that circ-ZNF609 is upregulated in biopsies from the two major RMS subtypes, embryonal (ERMS) and alveolar (ARMS). Moreover, we discovered that in an ERMS-derived cell line circ-ZNF609 knock-down induced a specific block at the G1-S transition, a strong decrease of p-Akt protein level and an alteration of the pRb/Rb ratio. Regarding p-Akt, we were able to show that circ-ZNF609 acts by counteracting p-Akt proteasome-dependent degradation, thus working as a new regulator of cell proliferation-related pathways. As opposed to ERMS-derived cells, the circRNA depletion had no cell cycle effects in ARMS-derived cells. Since in these cells the p53 gene resulted downregulated, with a concomitant upregulation of its cell cycle-related target genes, we suggest that this could account for the lack of circ-ZNF609 effect in ARMS.

## Introduction

CircRNAs are eukaryotic transcripts produced through a peculiar splicing reaction (back-splicing), which causes the circularization of one or more exons. The resulting molecules are characterized by a back-splicing junction and lack 5ʹ and 3ʹ free termini [1, 2]. Having such a shape, circRNAs are typically very stable molecules [3], a trait which has captivated a broad interest concerning their possible molecular activity. To date, despite the large number of identified circRNAs, the study of their mechanism of action remains largely unexplored. Moreover, quite diverse functions have been described so far for the few species studied: some molecules were shown to bind microRNAs, possibly acting as decoys or modulating their activity and localization [2, 4–7], while others were reported as RNA binding protein scaffolds or decoys [8, 9] or even to have protein coding ability [10–12].

So far, many circRNAs have been shown to be involved in cell cycle progression and several studies have focused on their role in abnormal proliferation of cancer cells. Overall, it seems that circRNAs could be a promising class of molecules for the better understanding of cancer progression, and thus have been studied in different cancer types [13–16]. Moreover, Guarnerio et al. [17]. showed that fusion-circRNAs, derived from tumor-associated chromosomal translocations, sustain cell proliferation and tumor aggressiveness.

We recently reported a knock-down based screening of circRNAs expressed in human myogenesis [10]. The knock-down of 25 targets followed by a detailed phenotypic characterization yielded one interesting molecule, circ-ZNF609, expressed in growing myoblasts and whose depletion resulted in the alteration of cell proliferation.

In this paper, we focus on the biological function of circ-ZNF609, describing its impact on myoblasts gene expression. Furthermore, because of circ-ZNF609 ability to promote myoblast proliferation, we studied its expression, function, and effects on the transcriptome in rhabdomyosarcoma (RMS), a pediatric skeletal muscle malignancy.

RMS accounts for roughly 5% of all pediatric tumors and it is the most common soft tissue sarcoma occurring in childhood [18]. It is a heterogeneous tumor, originating from mesenchymal precursor cells which express skeletal muscle markers but fail to complete a proper differentiation program [19, 20]. In children, RMS is conventionally classified in two main histological subtypes, the embryonal rhabdomyosarcoma (ERMS), more frequent and precocious, and the alveolar rhabdomyosarcoma (ARMS), which is associated to specific genetic alterations and has a generally worse prognosis due to its scarce response to treatment [18, 19]. Here we report that circ-ZNF609 is up regulated in RMS biopsies and cell lines. Furthermore, its depletion globally affects genes related to cell cycle and cell division in the ERMS subtype, blocking cell proliferation at the G1-S cell cycle checkpoint.

## Results

### Circ-ZNF609 depletion affects cell cycle genes and triggers immune response genes

**Fig. 1:**
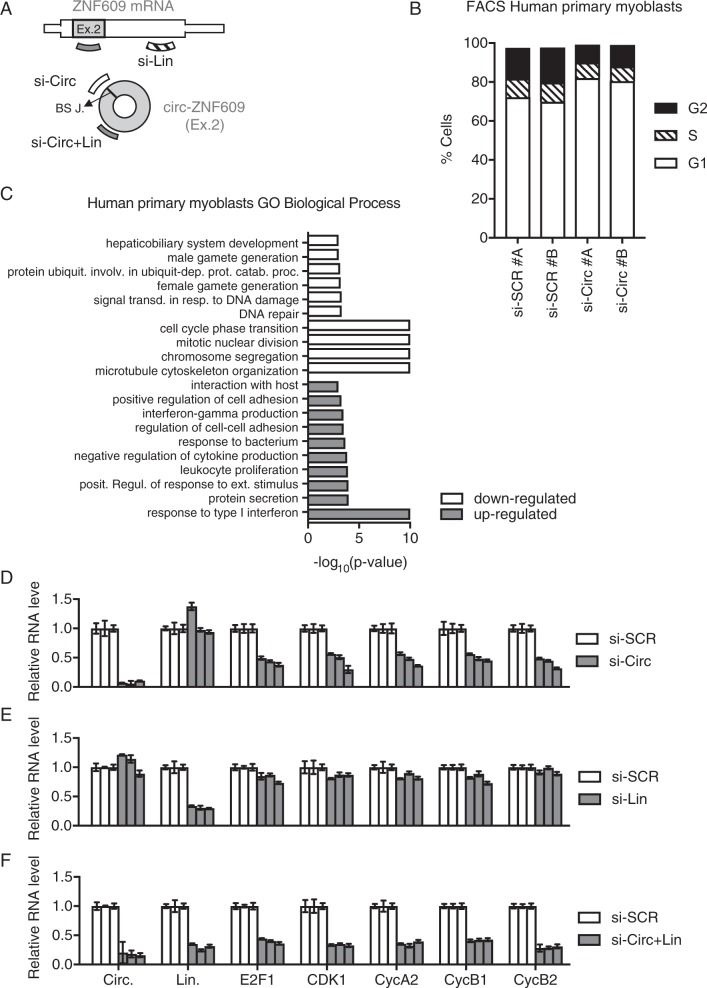
**a** Schematic representation of the siRNAs used against circ-ZNF609 and/or its linear counterpart, ZNF609 mRNA. Ex.2 = Exon 2; BS J. = back-splicing junction. **b** Cell cycle analysis by flow cytometry (FACS) of human primary myoblasts in growth conditions upon control treatment (si-SCR) or circ-ZNF609 knock-down (si-Circ). *N* = 2. **c** Gene Ontology (GO) term enrichment analysis (Biological Process) performed on genes down regulated (white) and up regulated (gray) upon circ-ZNF609 depletion in human primary myoblasts in growth conditions. **d**–**f** RNA levels measured by qRT-PCR of circ-ZNF609 (Circ.), ZNF609 (Lin.) and some genes involved in cell cycle (E2F1, CDK1, Cyclin A2, Cyclin B1, Cyclin B2) in si-SCR and either si-Circ (**d**), or si-Lin (**e**), or si-Circ + Lin (**f**), in human primary myoblasts in growth conditions. Data are shown as means ± standard error of technical duplicates. *N* = 3

To study circ-ZNF609 impact on gene expression, we treated human primary myoblasts with a control siRNA (si-SCR) or with an siRNA targeting the back-splicing junction (Fig. 1a, si-Circ) [10], in such way that the circRNA can be specifically silenced without affecting its linear counterpart (Figure S1A). FACS analysis indicated that, in comparison to a control siRNA (si-SCR), the specific knock-down of circ-ZNF609 induced a consistent arrest of proliferation (Fig. 1b). Then, transcriptomes of human primary myoblasts from two independent knock-down experiments were studied by means of ribosomal depleted total RNA sequencing (RNA-Seq). Gene expression-based clustering of samples showed that control siRNA-treated cells and specific siRNA-treated cells have clearly distinct expression profiles (Figure S1B). Differential expression analysis revealed 204 and 116 genes, respectively down regulated and up regulated in circ-ZNF609-depleted cells (adjusted *p*-value < 0.05, absolute log_2_ fold change > 0.5). These genes are listed in Table S1.

We then performed a functional enrichment analysis of downregulated and upregulated genes. The Biological Process GO terms enriched among the downregulated genes were mostly associated with cell cycle and mitosis, in agreement with the cell cycle arrest observed upon circ-ZNF609 depletion (Fig. 1c). On the other hand, an interesting association between the upregulated genes and GO terms related to the innate immune response and the interferon (IFN) signaling pathway (Fig. 1c) was also detected. However, IFN genes were not altered in their expression (Table S2), thus suggesting that the activation of the downstream genes can be independent from the IFN signaling and may be directly involved in cell cycle regulation, as already reported [21–24].

We used qRT-PCR to validate a set of differentially expressed genes in three biological replicates of the knock-down experiment (Fig. 1d). In particular, we analyzed E2F1, CDK1, cyclin A2 (CycA2), cyclin B1 (CycB1), and cyclin B2 (CycB2), which are related to the cell cycle. These data were consistent with the RNA-Seq results.

To exclude possible off-target effects due to non-specific knock-down of the ZNF609 mRNA, as well as other unrelated targets, we validated the modulation of the selected genes using two additional siRNAs: one targeting the linear mRNA (si-Lin) and the other targeting both isoforms (si-Circ+Lin) (Fig. 1a). As shown in Fig. 1e, f, si-Lin did not affect the genes modulated by si-Circ, while si-Circ+Lin did, hence indicating that E2F1, CDK1, CycA2, CycB1, and CycB2 expression is specifically responsive to circ-ZNF609 knock-down.

### Circ-ZNF609 is upregulated in RMS

**Fig. 2:**
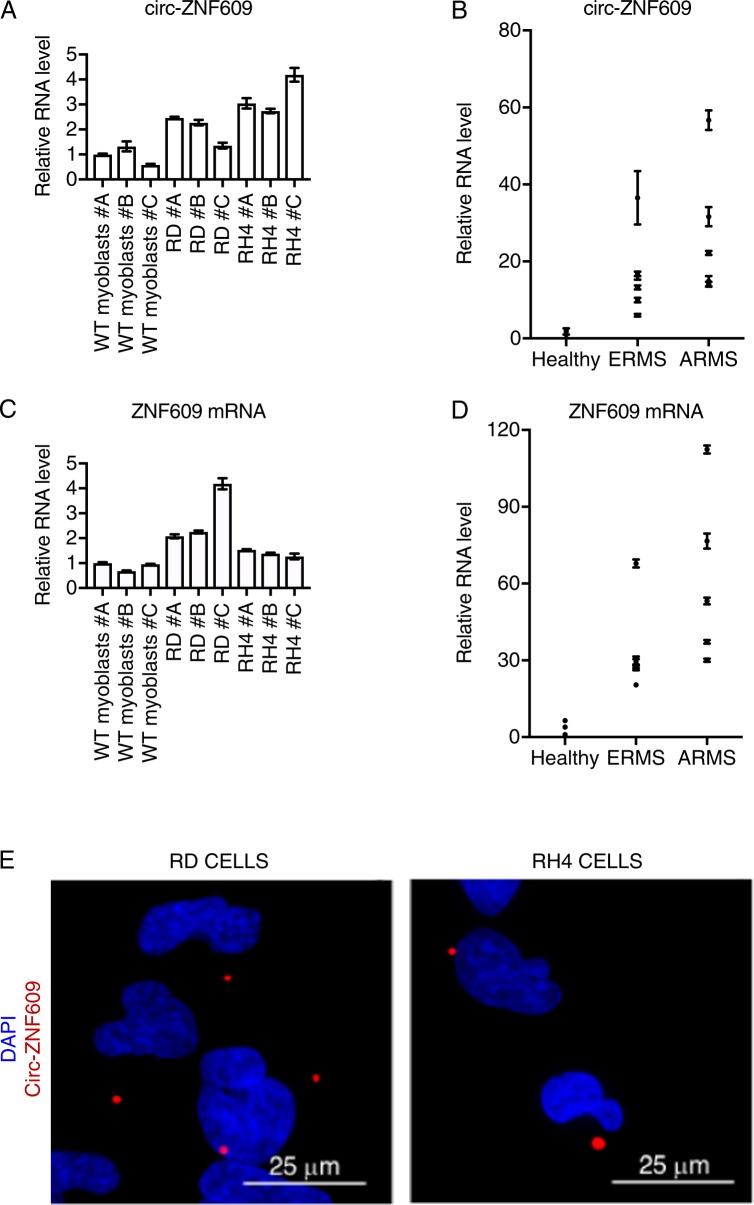
**a** RNA levels measured by qRT-PCR of circ-ZNF609 in wild-type human primary myoblasts (WT), RD cell line and RH4 cell line. Data are shown as means ± standard error of technical duplicates. *N* = 3. **b** RNA levels measured by qRT-PCR of circ-ZNF609 in healthy skeletal muscle (Healthy, *N* = 3), embryonal RMS (ERMS, *N* = 6) and alveolar RMS (ARMS, *N* = 5) samples from pediatric age-matched patients. Data are shown as means ± standard error of technical triplicates. **c** RNA levels measured by qRT-PCR of ZNF609 in wild-type human primary myoblasts (WT), RD cell line and RH4 cell line. Data are shown as means ± standard error of technical duplicates. *N* = 3. **d** RNA levels measured by qRT-PCR of ZNF609 in healthy skeletal muscle (Healthy, *N* = 3), embryonal RMS (ERMS, *N* = 6) and alveolar RMS (ARMS, *N* = 5) samples from pediatric age-matched patients. Data are shown as means ± standard error of technical triplicates. **e** FISH in RD cells (left) and RH4 cells (right) using a circ-ZNF609-specific probe

Since circ-ZNF609 is involved in myoblast proliferation, we focused our attention on RMS, a solid malignant tumor of the skeletal muscle. We initially measured circ-ZNF609 expression in wild-type (WT) myoblasts versus RMS cell lines (RD and RH4 cells, two in vitro models of ERMS and ARMS subtypes, respectively) and found a consistent upregulation of the circRNA in both the tumor cell lines, with particularly higher levels in the alveolar type (Fig. 2a). These observations may suggest that circ-ZNF609 plays a role in sustaining the aberrant proliferation rate of RMS cells.

We then looked at circ-ZNF609 expression in primary tumor biopsies obtained from 11 patients with RMS (6 ERMSs and 5 ARMSs), compared with skeletal muscle biopsies from three age-matched healthy donors. We found that circ-ZNF609 RNA levels are 5 to ∼50-fold higher in RMS tumors (Fig. 2b).

When looking at the ZNF609 linear mRNA counterpart, we found a similar pattern: up regulation in both RMS cell lines (Fig. 2c), and in RMS tumors (Fig. 2d). These findings suggest that the mechanism underlying such altered expression is likely related to the transcriptional activation of the gene.

We also checked circ-ZNF609 subcellular localization by fluorescent in situ hybridization (FISH), using a probe targeting the back-splicing junction of the circRNA. We performed this experiment in RD and RH4 RMS cell lines, and we observed that in both types of cells circ-ZNF609 localizes in specific cytoplasmic spots (Figs. 2e, S1C, D). The specificity of this signal was proven by its disappearance when cells were treated with si-Circ (Fig. S1E). As control, the use of a probe specific for the linear counterpart indicated a higher number of cytoplasmic spots (Fig. S1F, left). Finally, a bacterial-specific probe provided no signal (Fig. S1F, right). These results suggest that the circ-ZNF609 molecules are localized in a specific cytoplasmic sub-compartment. The FISH method utilized (see supplementary material) has a single-molecule detection sensitivity, however we believe that many of the identified spots could contain multiple copies of circ-ZNF609. This is mainly suggested by the different diameter of the spots (Fig. S1C, asterisks); unfortunately, due to the signal amplification procedure as well as to the optical microscope resolution limitations, at the moment it is impossible to exactly establish the number of copies inside each spot.

### Circ-ZNF609 depletion causes ERMS (but not ARMS) proliferation arrest in vitro

**Fig. 3:**
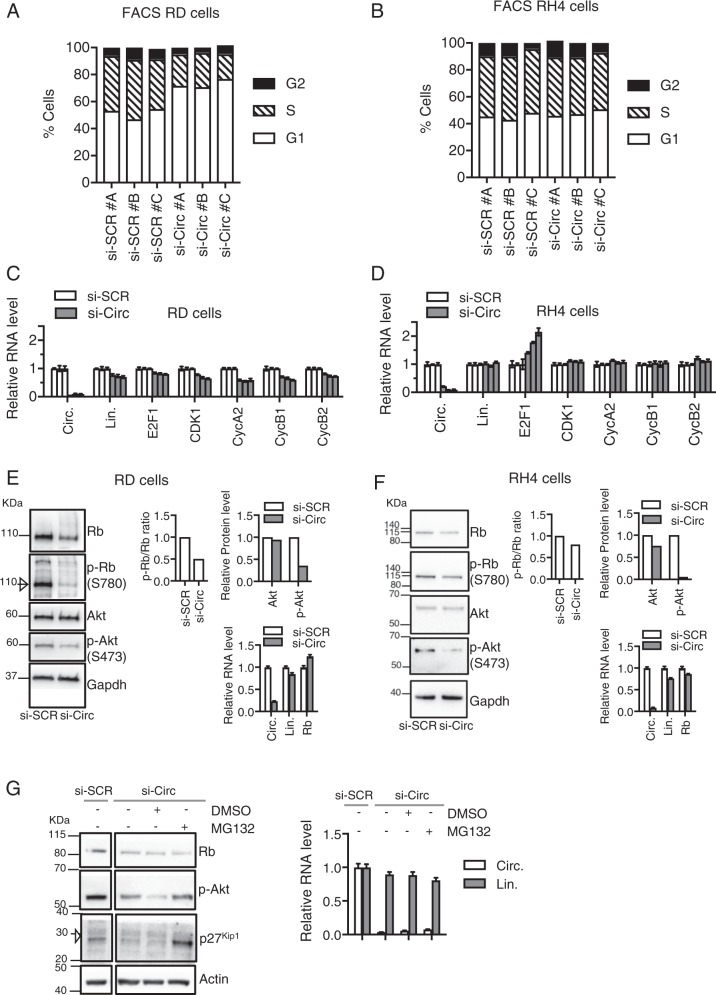
**a** Cell cycle analysis by flow cytometry (FACS) of RD cells upon control treatment (si-SCR) or circ-ZNF609 knock-down (si-Circ). *N* = 3. **b** Cell cycle analysis by flow cytometry (FACS) of RH4 cells upon control treatment (si-SCR) or circ-ZNF609 knock-down (si-Circ). *N* = 3. **c**, **d** RNA levels measured by qRT-PCR of circ-ZNF609 (Circ.), ZNF609 (Lin.) and some genes involved in cell cycle (E2F1, CDK1, Cyclin A2, Cyclin B1, Cyclin B2) in si-SCR or si-Circ conditions in RD cells (**c**) and in RH4 cells (**d**). Data are shown as means ± standard error of technical duplicates. *N* = 3. **e** Western blot in RD cells in si-SCR and si-Circ conditions (Gapdh hybridization was used as loading control). Corresponding ratio of pRb/Rb protein levels, Akt and p-Akt protein levels (relative to Gapdh) and RNA levels (measured by qRT-PCR of circ-ZNF609 (Circ.), ZNF609 (Lin.) and Rb) are shown. qRT-PCR data are shown as means ± standard error of technical duplicates. The experiment was repeated at least three times (*N* = 3). **f** Western blot in RH4 cells in si-SCR and si-Circ conditions (Gapdh hybridization was used as loading control). Corresponding ratio of pRb/Rb protein levels, Akt and p-Akt protein levels (relative to Gapdh) and RNA levels (measured by qRT-PCR of circ-ZNF609 (Circ.), ZNF609 (Lin.) and Rb) are shown. qRT-PCR data are shown as means ± standard error of technical duplicates. The experiment was repeated at least three times (*N* = 3). **g** Proteasome inhibition experiment in RD cells treated with either si-SCR or si-Circ: western blot and RNA analysis by qRT-PCR of circ-ZNF609 (Circ.) and ZNF609 (Lin.). qRT-PCR data are shown as means ± standard error of technical duplicates. The experiment was repeated at least three times (*N* = 3)

To study the biological role of circ-ZNF609 in RMS, we treated RD and RH4 cell lines with either si-SCR or si-Circ, and analyzed the effects on cell cycle progression. si-Circ induced a strong and reproducible block of the G1/S transition in RD cells (Figs. 3a and S2A), whilst no effect on the cell cycle was observed upon depletion of circ-ZNF609 in RH4 cells (Figs. 3b and S2B). The use of si-Circ+Lin and si-Lin proved that the effects observed in RD cells were specifically due to the circular form (Fig. S2C). Moreover, the genes modulated after circ-ZNF609 depletion in myoblasts (E2F1, CDK1, CycA2, CycB1, and CycB2) were found to be similarly affected in RD (Fig. 3c), but not in RH4 cells (Fig. 3d), with the exception of E2F1, whose levels were consistently upregulated (Fig. 3d). These findings demonstrate a relevant difference between the two RMS subtypes that could account for the more aggressive phenotype of ARMS cells and for their insensitivity to circ-ZNF609 knock-down.

Since in RD cells we observed the G1-S proliferation block and the down regulation of E2F1-targets, such as CDK1, CycA2, CycB1, CycB2 (Fig. 3c), PCNA and some DNA polymerase subunits (see RNA-Seq data discussed below and Table S1) [25–28], we decided to investigate some well-known protein factors regulating G1-S transition and cell proliferation. In particular, we focused our attention on the Retinoblastoma (Rb) and Akt proteins. Interestingly, in both RD and RH4 cells, and despite their different phenotypic response to circ-ZNF609 knock-down, these cell cycle-related proteins were found to respond to circ-ZNF609 depletion in a similar manner.

The Rb protein is a known regulator of the G1-S cell cycle checkpoint by controlling E2F1, a transcription factor that induces the expression of the S-phase genes. In particular, hypo-phosphorylated Rb can bind E2F1, therefore blocking its ability to activate S-phase genes and so causing G1-S arrest. In RD cells, a reduction of the total amount of Rb protein was observed, despite no changes in its mRNA level (Figs. 3e and S2D); moreover, protein decrease was accompanied by a strong decrease of its phosphorylated form, leading to a ratio between p-Rb and Rb down to approximately 50% compared to control conditions (Figs. 3e and S2D). These findings agree with data indicating that E2F1 targets are downregulated upon circ-ZNF609 depletion (Fig. 3c and Table S1). Therefore, the reduced p-Rb/Rb ratio, together with a slightly reduced E2F1 expression (Fig. 3c) upon circ-ZNF609 depletion, can likely induce the G1-S block. Also in RH4 cells, the levels of total Rb and of p-RB decreased after the circRNA depletion, despite an unaltered mRNA level (Figs. 3f and S2E); however, in these cells the pRb/Rb ratio decreased, but not reproducibly (Figs. 3f and S2E). Notably, the phosphorylated Akt (p-Akt), which has a crucial role in sustaining cell proliferation [29], was reduced upon circ-ZNF609 depletion in both RMS cell lines, while total Akt levels remained unaffected (Figs. 3e, f and S2D–E).

In conclusion, these experiments indicate that, even if the depletion of circ-ZNF609 has no effect on the proliferative features of RH4 cells, it maintains the specificity of action by targeting specific regulators of the cell cycle.

To investigate whether the decrease in Rb and p-Akt protein levels upon circ-ZNF609 knock-down was due to an increased protein degradation, we treated RD cells with a proteasome inhibitor (MG132) upon si-SCR or si-Circ transfection. As a positive control for proteasome inhibition we used p27^Kip1^ [30]. We observed that Rb protein levels did not increase upon proteasome inhibition, whereas p-Akt accumulated in si-Circ upon MG132 treatment (Figs. 3g and S2F).

### Circ-ZNF609 knock-down induces different transcriptomic responses in ERMS and ARMS

**Fig. 4:**
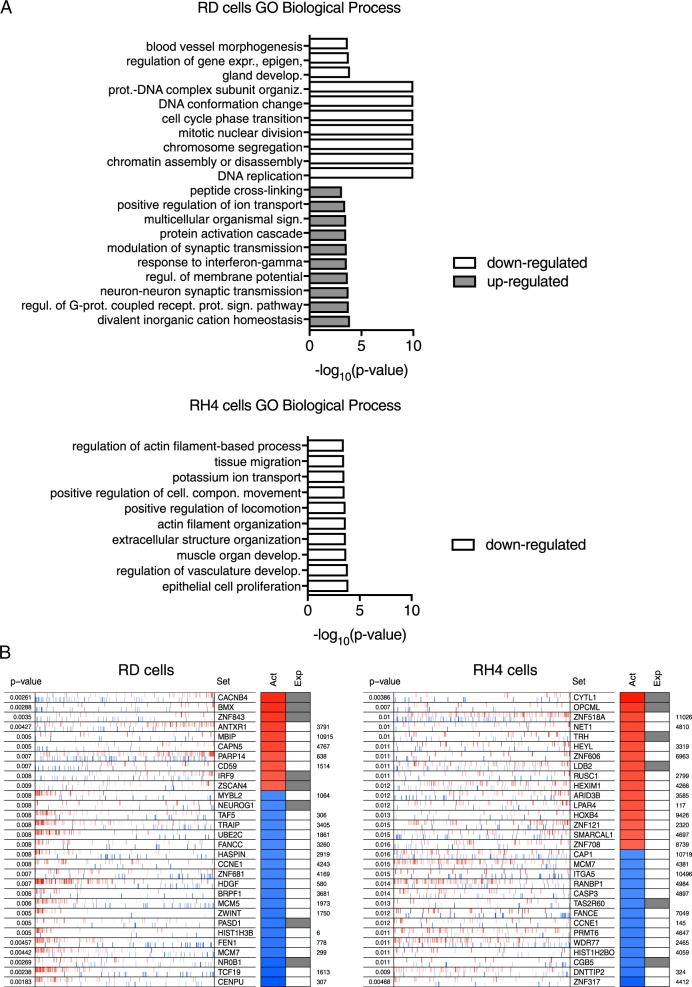
**a** Gene Ontology (GO) term enrichment analysis (Biological Process) performed on genes downregulated (white) and upregulated (gray) upon circ-ZNF609 depletion in RD cells (upper panel) and RH4 cells (lower panel). For RH4 cells, there was not any enriched category for upregulated genes. **b** msVIPER analysis plot for RD cells (left) and RH4 cells (right) showing the transcriptional status (blue = repressed, red = activated) of the target genes for each differentially active transcriptional regulator (genes are represented by vertical bars in each horizontal row), drawn using the *plot.msviper* function. The right side of the figure displays the inferred differential activity (first column, red = activated and blue = de-activated) and expression (second column, gray = not expressed and white = expressed), with the rank of the displayed genes in the VIPER inferred gene expression signature (numbers at the right side of the plot). *p*-values refer to the differential activity of the factor calculated by msVIPER

Since circ-ZNF609 knock-down has different effects depending on the RMS subtype, we decided to investigate and compare transcriptomic changes following its depletion in ERMS and ARMS cell lines. We performed RNA-Seq on two replicates of RD cells and RH4 cells in control (si-SCR) and circ-ZNF609 knock-down (si-Circ) conditions (Fig. S2G). As shown in Fig. S2H, gene expression-based clustering of samples correctly recapitulates cell identities. After performing a differential expression analysis, we investigated the GO terms significantly associated to genes downregulated and upregulated in circ-ZNF609 depleted cells (lists of differentially expressed genes are reported in Table S1). As expected, in RD cells the Biological Process GO terms enriched among the downregulated genes were mostly associated with cell cycle progression, DNA replication and mitosis, whereas amongst the upregulated genes we found different categories, including “Response to interferon-gamma” (Fig. 4a, upper panel). On the contrary, RH4 cells did not show any enriched category strictly related to cell cycle regulation among downregulated genes, and any enriched category among the upregulated ones (Fig. 4a, lower panel).

We then performed a GGEA (Gene Graph Enrichment Analysis) [31] to compute consistency of the observed differential expression data with the regulatory interactions known for different KEGG pathways [32]. Among the most significant and consistent results for RD cells there were numerous pathways related to cell cycle, such as “DNA replication”, “Cell cycle” and “PI3K-Akt signaling pathway” (Figs. S3A and Table S3). On the other hand, in RH4 cells less significant results were obtained, among which the most interesting were represented by TGF-beta, Hippo and PI3K-Akt signaling pathways (Figs. S3B and Table S3).

To investigate the upstream regulators of these transcriptomic responses in RD and RH4 cells upon circ-ZNF609 knock-down, we performed msVIPER (Virtual Inference of Protein-activity by Enriched Regulon) analysis [33] in order to determine which protein factors could be responsible for the transcriptional alteration of the different regulons involved in cell proliferation. In RD cells, msVIPER analysis indicated TCF19, CENPU, MCM5–7 and ZWINT among the transcription factors whose activity was repressed (Fig. 4b, left). Interestingly, these genes were down regulated upon circ-ZNF609 depletion (Fig. S3C, left). They are also known to generally promote G1-S transition or chromosome segregation [34–41] and, in the case of TCF19 and MCMs, to be predicted or validated direct targets of E2F1 [27, 42–44].

Differently to what was observed in RD, in RH4 cells msVIPER results had higher p-values and a less obvious relationship to the cell cycle regulation (Fig. 4b, right). Notably, TCF19, CENPU, MCM7 and ZWINT expression levels were not affected upon circ-ZNF609 knock-down (Fig. S3C, right), even if expressed at lower levels than in RD cells (Fig. S3D, MCM7, CENPU, ZWINT: asterisks). Moreover, the expression levels of downstream cell-cycle related genes, many of which were even more highly expressed in RH4 than in RD cells, were not affected (Fig. S3D). Interestingly, a significant subset of cell cycle related genes up regulated in RH4 (CDK2, CDKN1B, E2F7, E2F1, ZNF385A, PCBP4, CCNA2, CCNA1, CCNE1, CCNE2) is composed of p53 targets (FDR = 4.02e−02 for the enrichment of Reactome [45] pathway “TP53 Regulates Transcription of Genes Involved in G1 Cell Cycle Arrest”). The p53 gene is mutated in both RD (gain-of-function R248W homozygous mutation) and RH4 cells (frameshift deletion of 1001–1013 nucleotides) [46–48]; moreover, p53 expression levels are significantly lower in RH4 than in RD cells (Table S1). These features might explain the significantly high expression of the p53 target genes involved in cell cycle regulation in RH4 cells as well as the lack of an effect of circ-ZNF609 depletion on the ARMS cell line.

Altogether, these results suggest that circ-ZNF609 can control a specific part of the transcriptome in RD cells, similarly to what is observed in human primary myoblasts, by inducing, when downregulated, a specific gene expression response related to the cell cycle and therefore fully consistent with the observed G1-S arrested phenotype. On the other hand, in RH4 cells, circ-ZNF609 depletion induces a less clear effect on the transcriptome, with very limited alterations of cell proliferation-related genes and on IFN response-related genes (Fig. S3E), hence likely reflecting the lower p53 levels.

## Discussion

Circular RNAs represent a recently re-discovered class of covalently closed RNAs, produced by all eukaryotic cell types and conserved among different species. A general mechanism of function has not been discovered, however their conservation, tissue-specificity and abundance suggest possible biological roles. Here we focused on circ-ZNF609 and on its role in regulating cell cycle progression in RMS. Through sequencing of RNA from human primary myoblasts depleted of circ-ZNF609, we discovered that this circRNA controls two well-defined classes of genes: cell cycle-related and innate immune response genes, that are respectively downregulated and upregulated. Notably, these opposing effects seem to suggest that these two classes of genes act on a common pathway of proliferation control. Indeed, since there is evidence that IFN stimulated genes induce cell cycle block [21–24], it is possible that the induction of the innate immune response genes can cooperate with the proliferative arrest observed upon circ-ZNF609 depletion.

To deepen our knowledge regarding circ-ZNF609 role in cell proliferation control, we studied its expression and function in RMS. Circ-ZNF609 resulted upregulated in cell lines and primary samples from both ERMS and ARMS tumors. RNA sequencing in the RD (ERMS) cell line upon circ-ZNF609 knock-down highlighted a similar gene expression response as in human primary myoblasts, with the downregulation of several genes involved in cell cycle control. Moreover, in RD cells the depletion of the circRNA induced a specific block at the G1-S transition. Searching for the factors and pathways possibly involved in such a block, we found that p-Rb/Rb ratio and p-Akt protein level were strongly decreased, whilst the level of the corresponding mRNAs was not affected significantly. In the case of p-Akt, we were also able to show that circ-ZNF609 counteracts p-Akt proteasome-dependent degradation, thus working as a new regulator of cell proliferation-related pathways. Notably, the transcriptional outcome of circ-ZNF609 depletion resulted in the downregulation of well-known genes directly targeted by Rb through E2F1 regulation: among them CycA2, CycB1, PCNA, some DNA polymerase subunits, and transcriptional regulators such as MCMs and TCF19. Of these factors, we identified TCF19, MCM7, together with CENPU and ZWINT, as the master regulators of G1-S transition which are deregulated upon circ-ZNF609 depletion.

As opposed to RD, in ARMS-derived RH4 cell lines, the depletion of the circRNA did not induce any block of cell proliferation and transcriptional analysis did not highlight any modulation of the aforementioned cell cycle related genes, even if the levels of Rb, p-Rb, and p-Akt proteins were affected in a similar manner to RD cells.

Searching for differences that could explain the variation between the behavior of the two RMS subtypes, we found a strong downregulation the p53 gene in RH4 cells; indeed, the analysis of p53 cell cycle-related target genes indicated that in these cells they are strongly up regulated when compared to RD. This difference could explain why circ-ZNF609 knock-down cannot induce proliferation arrest in ARMS-derived RH4 cells. ARMS is the most aggressive RMS subtype and many pathways involved in cell proliferation are altered in this tumor, meaning that the effects of circ-ZNF609 alone may not be sufficient for a significant reduction in the rate of cell growth.

We are very much intrigued by the characterization of the molecular mechanisms through which circ-ZNF609 affects pRb/Rb ratio and p-Akt protein levels, as well as the levels of G1-S transition master regulators. In particular, the translational control of Rb and the protection of p-Akt from proteasomal degradation represent the two most interesting and promising examples to highlight potential novel regulatory mechanisms mediated by circRNAs. We believe that the identification of the RNA and protein interactors of circ-ZNF609 (possible RNA-RNA interactions controlling protein translation or RNA-protein complexes mediating proteasomal activity) will be instrumental to distinguish between direct and indirect effects and to approach the clarification of its mechanisms of action as well as the link with the cell cycle phenotype.

The pathways described in our work seem to differ from what was recently described by Peng et al. [49] in Hirschsprung disease biopsies. In their case, circ-ZNF609 was proposed to inhibit cell proliferation by acting as a competing endogenous RNA for miR-150-5p, modulating the expression of its target Akt3. Indeed, we checked whether the Akt3 protein and RNA levels were affected by the downregulation of circ-ZNF609, but neither of them was downregulated (Fig. S3F). It is therefore likely that in our model system circ-ZNF609 is not acting through this circuitry.

In conclusion, by investigating the differences between RD and RH4 transcriptomic responses to circ-ZNF609 depletion we have contributed towards clarifying the pathways controlled by this molecule. Future studies in our laboratory will be aimed at the further elucidation of the mechanism through which the circRNA controls the Rb, p-Rb and p-Akt levels in order to gain further knowledge regarding the processes that correlate circ-ZNF609 levels with cell proliferation.

## Materials and methods

### Cell culture and transfections

Wild-type human primary myoblasts (Telethon Biobank) were obtained from a skeletal muscle biopsy from a 2-year-old male child. They were cultured in growth medium (GM): DMEM (Sigma-Aldrich, Saint Louis, MO, USA), 10% FBS (Sigma-Aldrich), l-glutammine (Sigma-Aldrich) 2 mM, insulin (Sigma-Aldrich) 50 mg/ml, FGFb (Millipore - Merck) 25 ng/ml, EGF (Corning, Corning, NY, USA) 1 ng/ml, penicillin-streptomycin 1 × (Sigma-Aldrich). Human ERMS RD cell line and ARMS RH4 cells line [50] were cultured in DMEM (Sigma-Aldrich) supplemented with 10% FBS (Sigma-Aldrich), l-glutammine (Sigma-Aldrich) 2 mM and penicillin-streptomycin (Sigma-Aldrich). RD and RH4 cell lines were the same as Megiorni et al. [51] used in their work, and they were previously authenticated as described in [51]. No information available about human primary myoblasts (Telethon Biobank) authentication. All cell lines were tested for mycoplasma contamination.

To transfect cells, 3 pmol siRNA (Dharmacon, Lafayette, CO, USA) and 0.15 μl Dharmafect transfection reagent 1 (Dharmacon) in 150 μl DMEM (Sigma-Aldrich) were added to each 100 μl of culture medium in the plate. The mixture was vortexed for 10 s, left at room temperature for 20 min and then seeded in the plate. Medium was replaced 24 h after the transfection. siRNAs used in this work (si-SCR, si-Circ, si-Lin, si-Circ+Lin) were described in [10], where they are named si-scr, si-circ, si-mRNA, and si-ex2 respectively.

### Patient biopsies

Tumor samples from 11 primary RMS tumors, 5 ARMSs and 6 ERMSs, were obtained at diagnosis before any treatment from children admitted to the Department of Oncology at Alder Hey Children’s NHS Foundation Trust, Liverpool, United Kingdom. Control RNA was extracted from normal skeletal muscle biopsies obtained from 3 children undergoing surgery for non-oncological conditions. Institutional written informed consent was obtained from the patient’s parents or legal guardians. The study underwent ethical review and approval according to the local institutional guidelines (Alder Hey Children’s NHS Foundation Trust Ethics Committee, approval number 09/H1002/88).

### Proteasome inhibition

Cells in 10 cm plates were transfected with si-SCR or si-Circ (Dharmacon) as previously described. Twenty-four hours after transfection, si-Circ-transfected cells were split into three 60 mm plates and the si-SCR-transfected cells were sub-cultured as well. After additional 24 h, si-SCR-transfected cells and one plate of si-Circ-transfected cells were harvested; fresh medium supplemented either with MG132 (10 μM, dissolved in DMSO, Sigma-Aldrich) or with an equal volume of 100% DMSO (Sigma-Aldrich) was added to the other two si-Circ-transfected plates. DMSO-treated and MG132-treated cells were harvested after 5 h of treatment. The experiment was performed in at least 3 biological replicates.

### Flow cytometric analysis of cell cycle

Cells were pelleted and 100 μl PBS (Sigma-Aldrich) and 10 μl RNase-A (Sigma-Aldrich) were added to the pellet (1 mg/ml). Cells were incubated at 37 °C for 30 min. Then propidium iodide (Sigma-Aldrich) was added (1 mg/ml) and cells were incubated at room temperature, in the dark, for 5 min. Samples were processed using a BD FACSCalibur Flow Cytometer (BD Biosciences, Franklin Lakes, NJ, USA) machine and Cell Quest Pro (BD Biosciences) software. Results were analyzed using ModFit 3.1 software (BD Biosciences). Biological replicates performed for each experiment were at least: 2 (Fig. 1b), 3 (Fig. 3a, b), 2 (Figure S2C).

### RNA isolation, treatments, and analysis

Total RNA was extracted with Qiazol reagent (Qiagen, Hilden, Germany) and Direct-zol RNA Miniprep kit (Zymo Research, Irvine, CA, USA), according to the manufacturer’s protocol. Reverse transcription reaction was performed using PrimeScript RT Reagent Kit (Takara Bio USA) on an appropriate amount of RNA (usually between 100 and 500 ng). cDNA was diluted to 0.7–1 ng/μl with bidistilled water and used for the qRT-PCR reaction. qRT-PCR was performed as follows: 6 μl cDNA (0.7–1 ng/μl) were added to 7.5 μl PowerUp SYBR Green Master Mix (Thermo Fisher Scientific) and 1.5 μl of a 5 μM primers mix. DNA amplification was achieved following the manufacturer’s protocol. An ABI 7500 Fast qPCR (Thermo Fisher Scientific) instrument was used for amplification and data analysis. RNA levels are relative to HPRT mRNA (in experiments performed in human primary myoblasts), GAPDH mRNA (in experiments performed in RD and RH4 cells), ACTB mRNA (in proteasome inhibition experiments). Oligonucleotide sequences are provided in Table S4. Primers for circ-ZNF609 and ZNF609 mRNA were described in [10]. Primers for IFN-A1/13, IFN-A10, IFN-A21 were described in the reference [52]. Biological replicates performed for each experiment were at least: 3 (Fig. 1d–f), 3 (Fig. 2a, c), 3 (Fig. 3c, d), 3 (Figure S3C). Regarding qRT-PCR plots, data are shown as means ± standard error of technical replicates (error bars).

### Western Blot

Cells were harvested with a suitable volume of Protein Extraction Buffer (100 mM Tris pH 7.5, EDTA 1 mM, SDS 2%, PIC 1× (Complete-EDTA free, Roche—Merck, Darmstadt, Germania), incubated 20 min on ice and centrifuged at 15,000×*g* for 15 min at 4 °C. Proteins (25–30 μg) were loaded on 4–12% bis-tris-acrylamide gel (Thermo Fisher Scientific-Life Technologies) and transferred to a nitrocellulose membrane. The membrane was blocked in 5% milk and hybridized with the following antibodies: anti-Gapdh (6C5, #sc-32233, Santa Cruz Biotechnology, Dallas, TX, USA), anti-Rb (G3-245, #554136, BD Biosciences), anti-phospho-Rb Ser780 (D59B7, #8180, Cell Signaling Technology, Danvers, MA, USA), anti-Akt (#9272, Cell Signaling Technology), anti-phospho-Akt Ser473 (#9271, Cell Signaling Technology), anti-p27^Kip1^ (C-19, sc-528, Santa Cruz Biotechnology), anti-Actin (#A3854, Sigma-Aldrich), anti-Akt3 (ab152157, Abcam, Cambridge, UK). Images were acquired using a ChemiDoc MP Imager (Bio-Rad, Hercules, CA, USA) and analyzed using Image Lab 5.2.1 software (Bio-Rad). Whole images were adjusted in contrast and brightness when necessary. Protein samples were run twice, to allow multiple hybridizations for proteins with the same molecular weight. Gapdh hybridization was performed for each running, but only one Gapdh hybridization is shown. However, for protein quantification each specific signal was compared with the corresponding Gapdh hybridization. Biological replicates performed for each experiment were at least: 3 (Fig. 3e and S2D), 3 (Fig. 3f and S2E), 2 (Fig. S3F).

## Supplementary information

Supplemental Material

Table S1

Table S2

Table S3

Table S4

Table S5

## Data Availability

RNA sequencing raw data have been deposited at Gene Expression Omnibus (GSE117609).
