# Supplementary material for: Circ-ZNF609 regulates G1-S progression in rhabdomyosarcoma
Source: Oncogene. 2019 Jan 22;38(20):3843–54. doi: 10.1038/s41388-019-0699-4 (PMC6544520; doi:10.1038/s41388-019-0699-4)
Supplement: Supplementary file 1 — Supplemental Material [file 41388_2019_699_MOESM1_ESM.pdf]

# Supplementary Figures

1

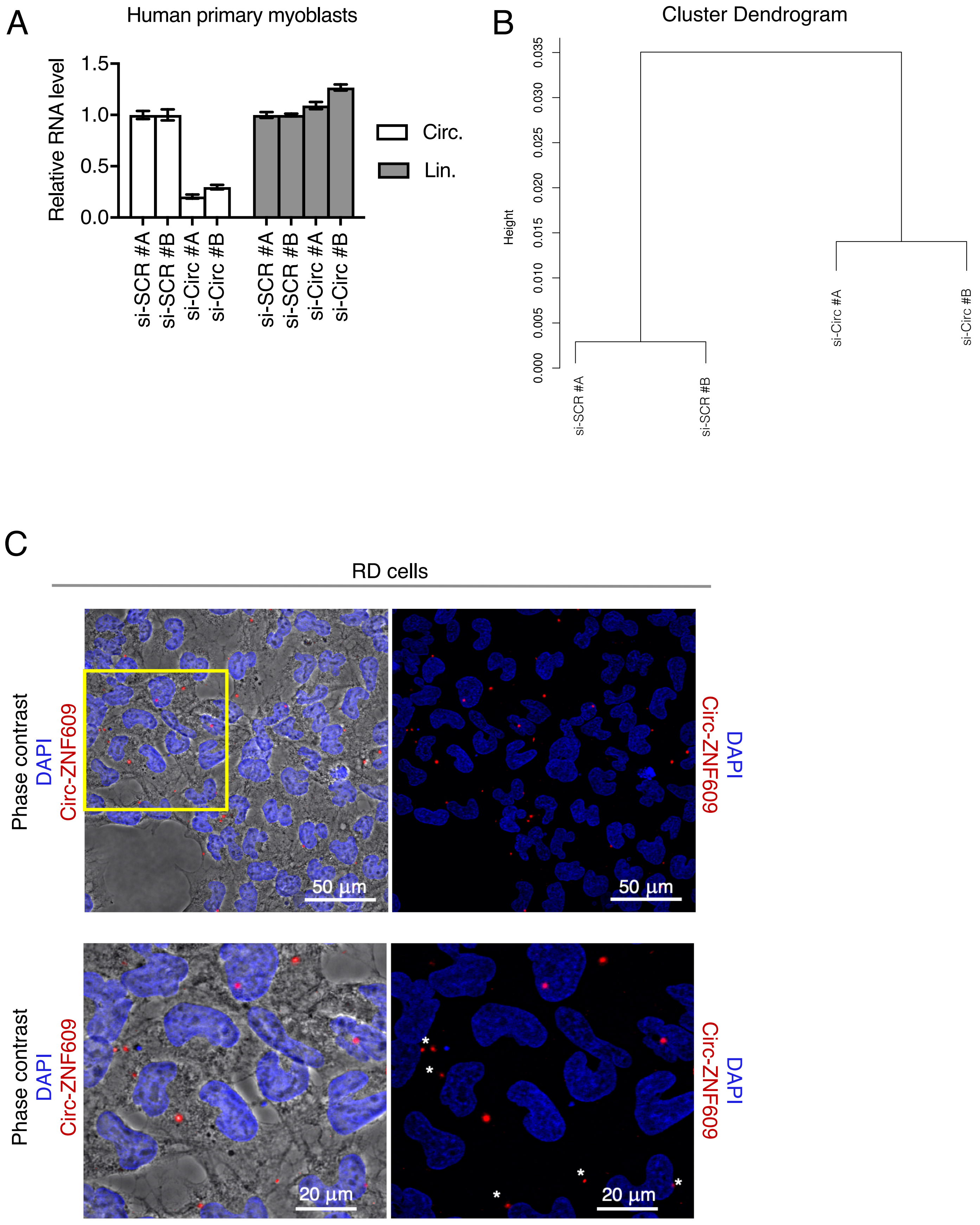

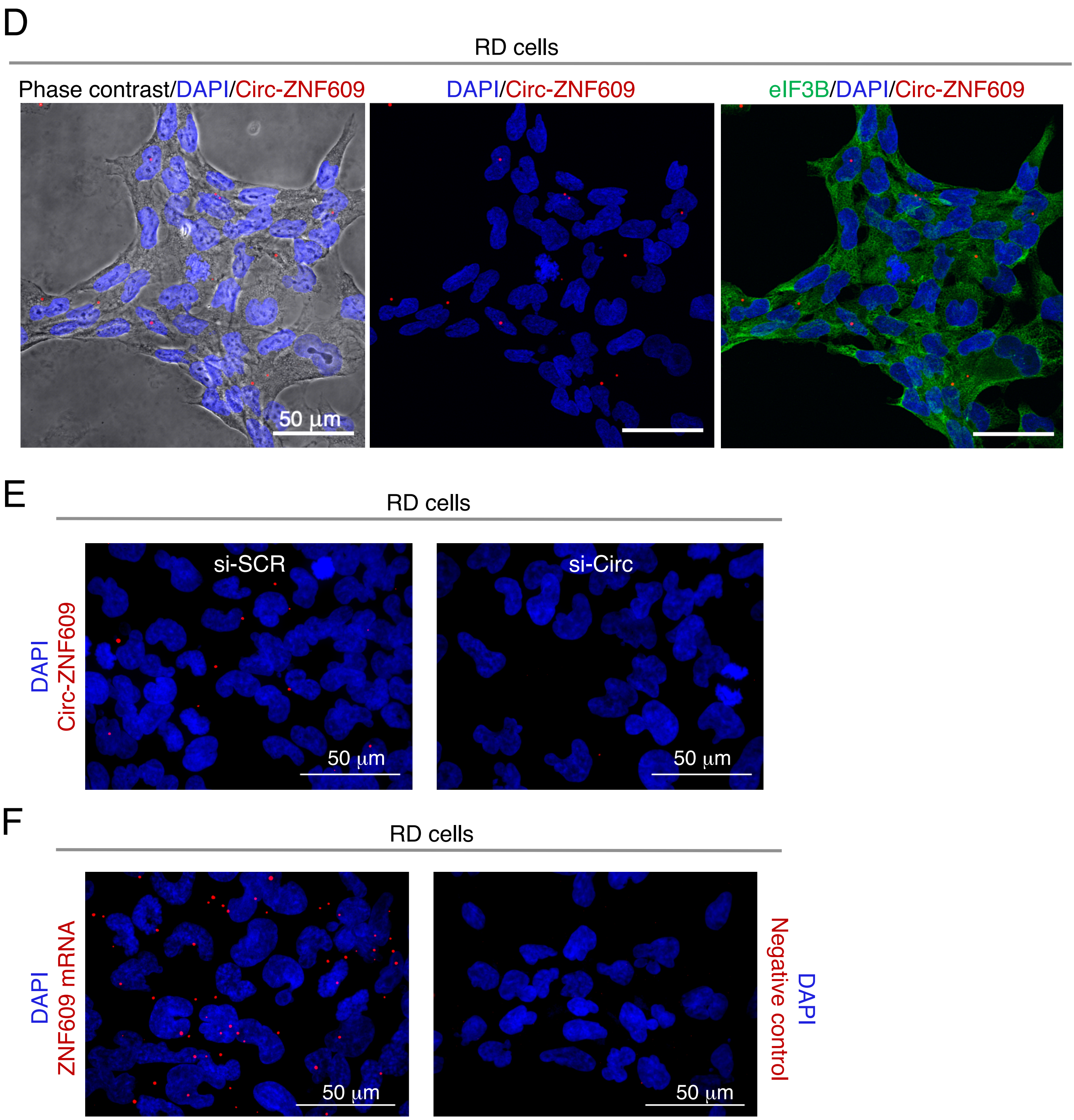


Figure S1

**A.** RNA levels measured by qRT-PCR of circ-ZNF609 (Circ.) and ZNF609 (Lin.) in human primary myoblasts treated either with control siRNA (si-SCR) or with si-Circ and sent for the RNA-seq analysis. Data are shown as means ± standard error of technical duplicates. N=2.
**B.** Hierarchical clustering of samples based on Pearson correlation of gene expression profiles in human primary myoblasts treated either with si-SCR (si-SCR #A and #B) or with si-Circ (si-Circ #A and #B).
**C.** FISH in RD cells using a circ-ZNF609-specific probe (red staining): on the left, merged image with DAPI staining, circ-ZNF609 staining and phase contrast to highlight cell borders; on the right, merged image with only DAPI staining and circ-ZNF609 staining. In the yellow square there is the image area zoomed in the lower panel. White asterisks indicate smaller circ-ZNF609 spots.
**D.** FISH using a circ-ZNF609-specific probe (red staining) combined to eIF3B immunofluorescence (green staining), in RD cells; left panel: merged image with DAPI staining, circ-ZNF609 staining and phase contrast; central panel: merged image with DAPI staining and circ-ZNF609 staining; right panel: merged image with DAPI staining, circ-ZNF609 staining and eIF3B staining.
**E.** FISH in RD cells using a circ-ZNF609-specific probe (red staining), in si-SCR and si-Circ.
**F.** FISH in RD cells using either a ZNF609-specific probe (left) or a bacterial-specific probe (“negative control”, right).

# Supplementary Figures

2

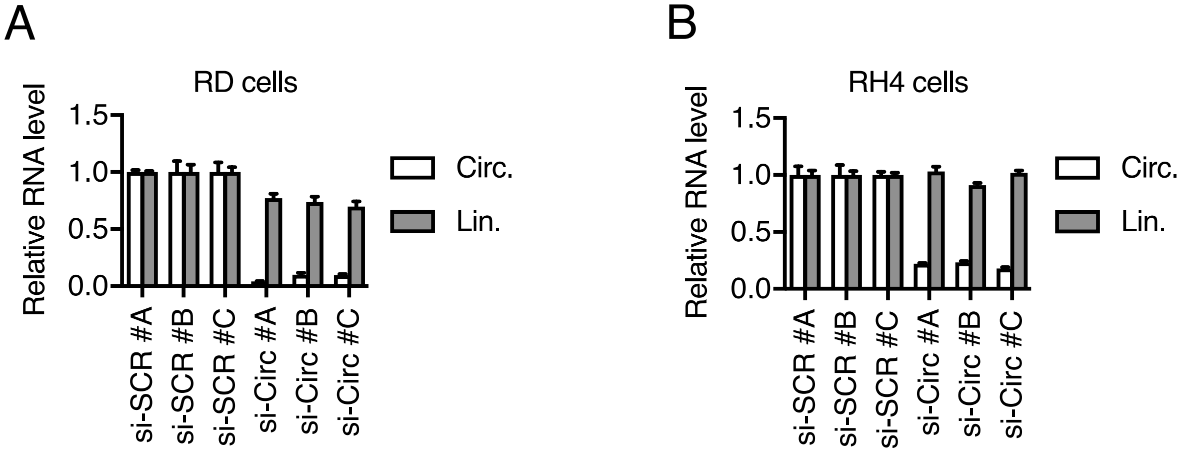


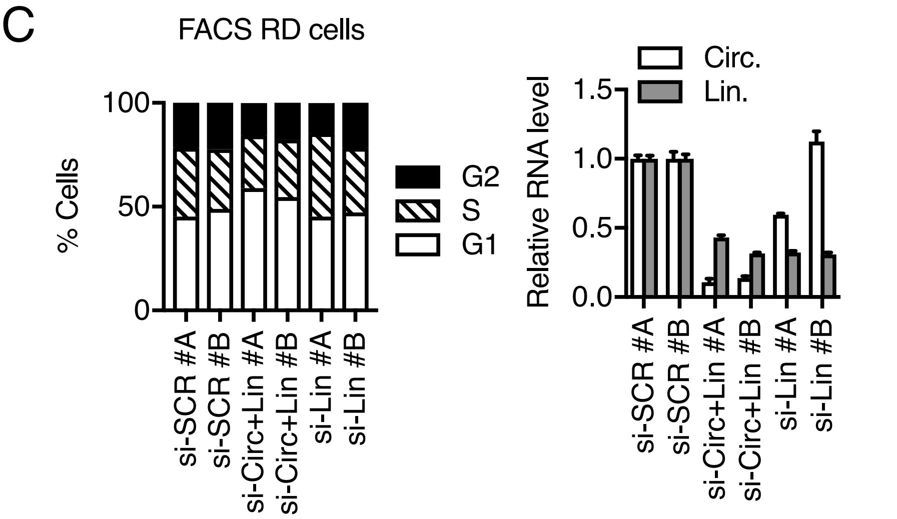


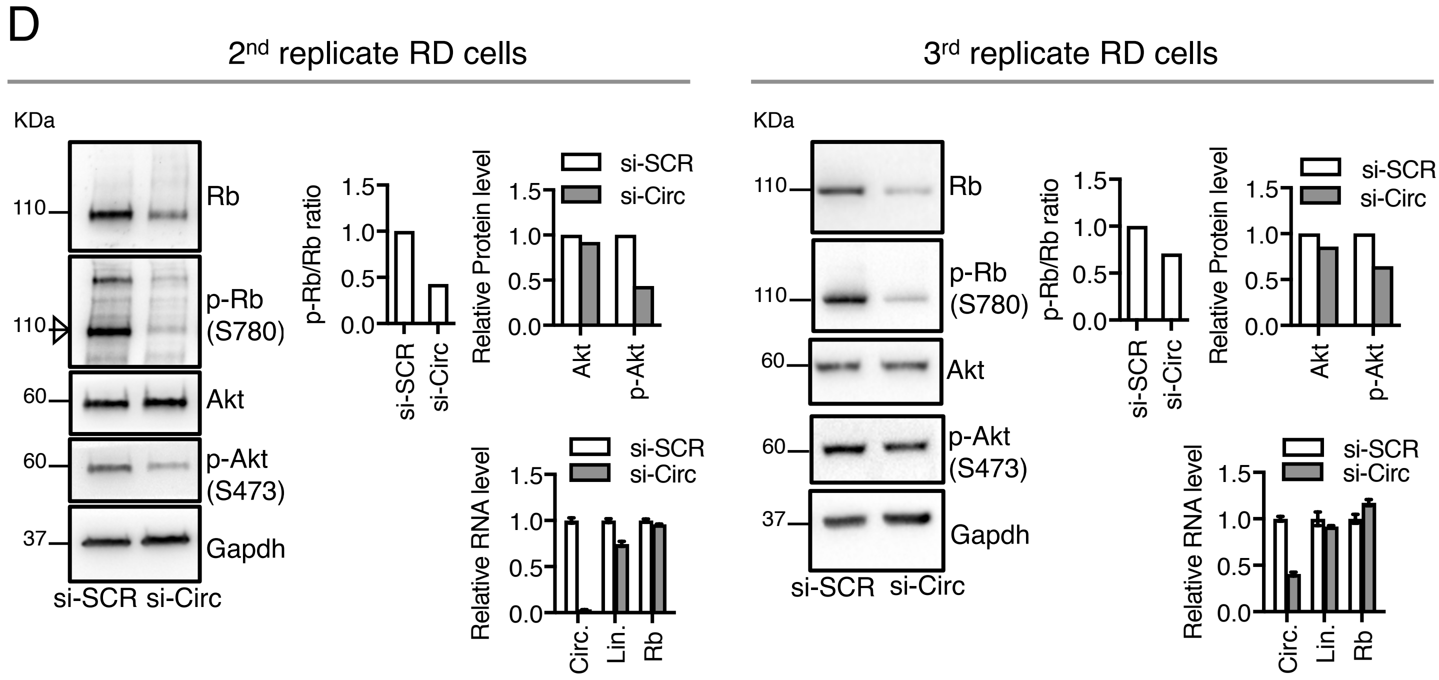

E

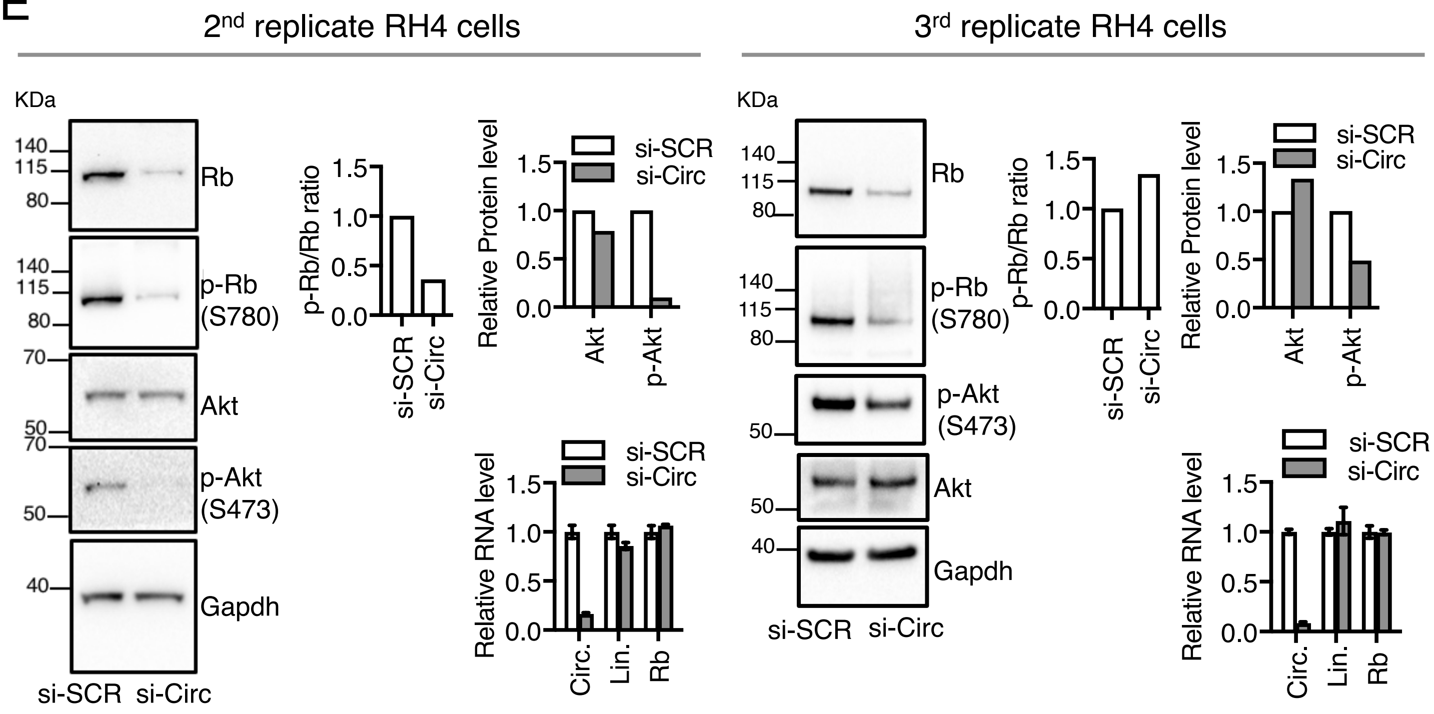

2nd replicate RH4 cells
3rd replicate RH4 cells
KDa
Rb
p-Rb (S780)
Akt
p-Akt (S473)
Gapdh
si-SCR si-Circ
p-Rb/Rb ratio
Relative Protein level
Relative RNA level
si-SCR
si-Circ
Circ.
Lin.
Rb


F

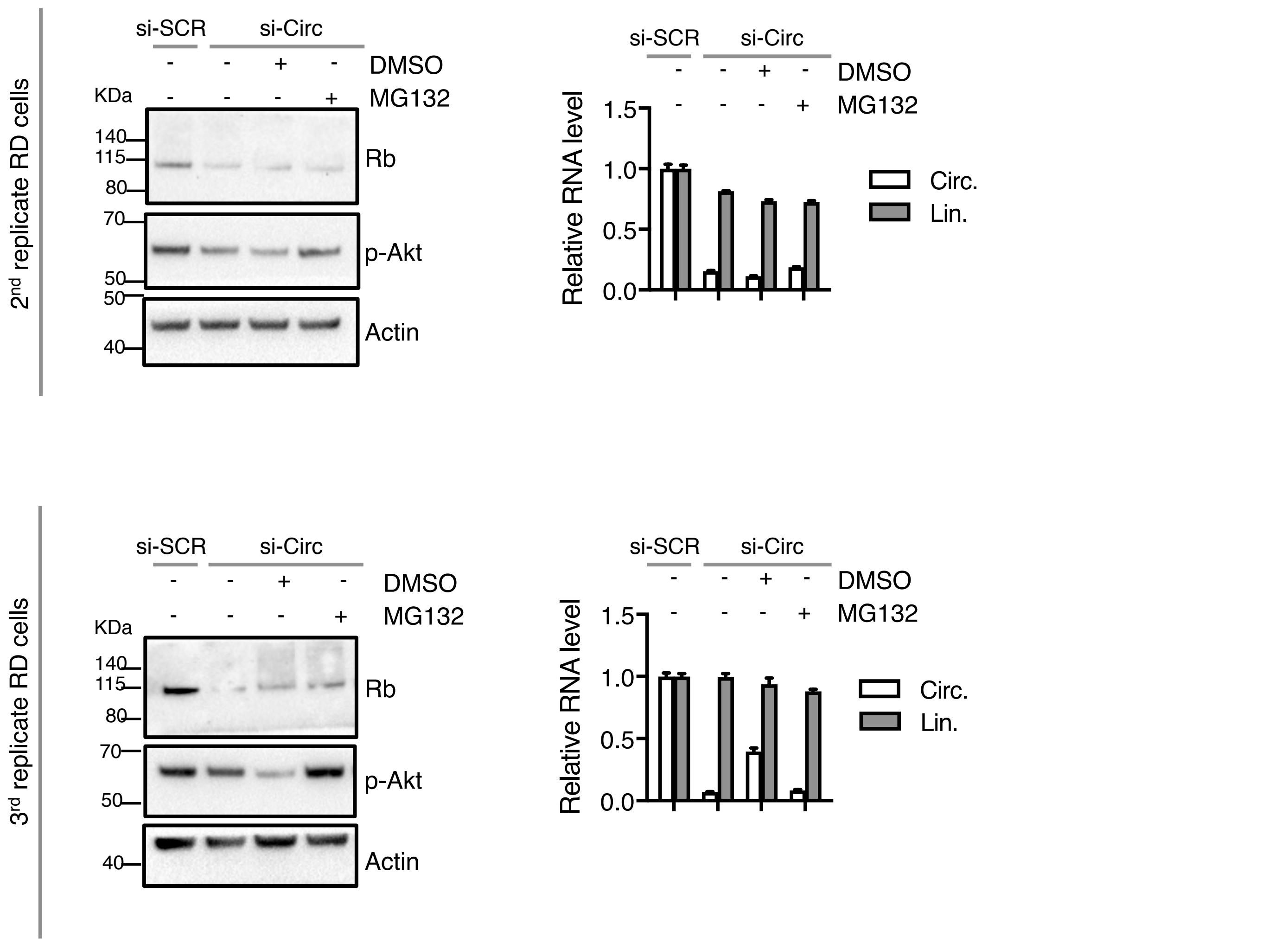

2nd replicate RD cells
3rd replicate RD cells
si-SCR
si-Circ
DMSO
MG132
KDa
Rb
p-Akt
Actin
Relative RNA level
Circ.
Lin.

G

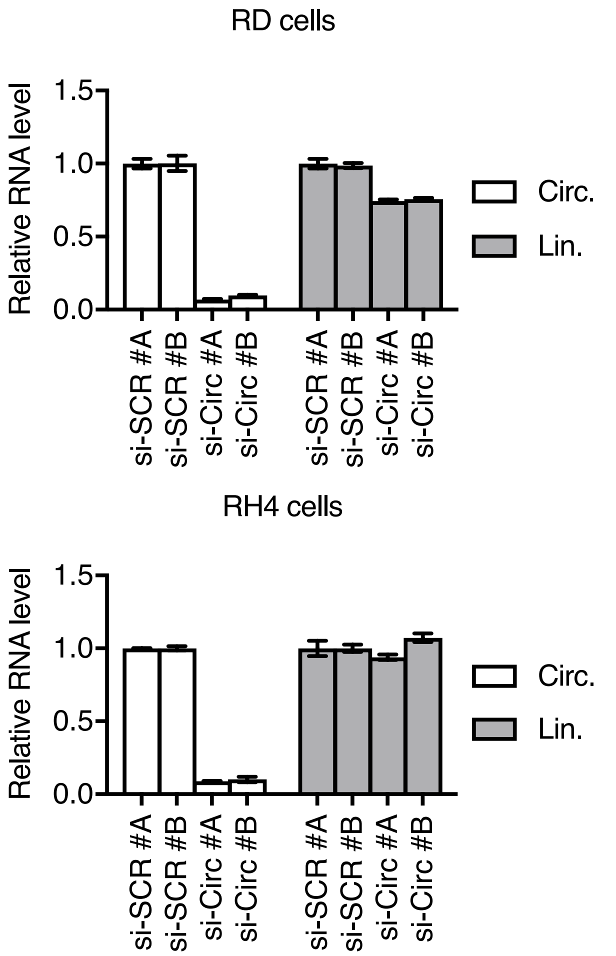


H

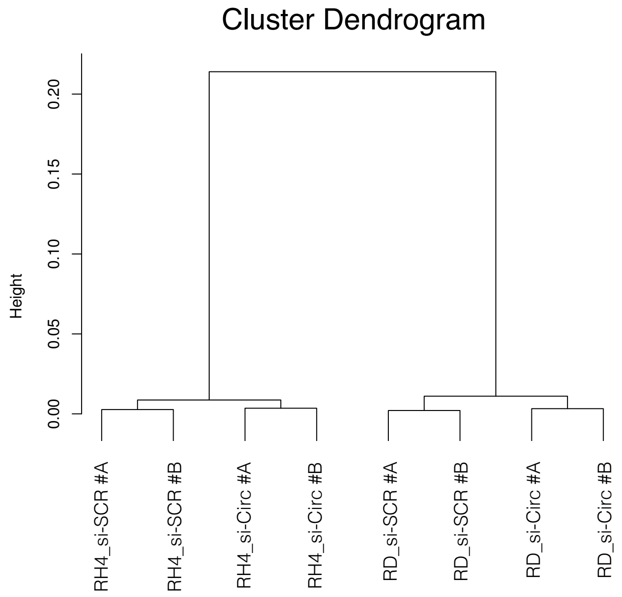


Figure S2

**A.** RNA levels measured by qRT-PCR of circ-ZNF609 (Circ.) and ZNF609 (Lin.) in three replicates (#A, #B and #C) of RD cells treated with either si-SCR or si-Circ and analyzed for FACS cell cycle analysis. Data are shown as means ± standard error of technical duplicates. N=3.

**B.** RNA levels measured by qRT-PCR of circ-ZNF609 (Circ.) and ZNF609 (Lin.) in three replicates (#A, #B and #C) of RH4 cells treated with either si-SCR or si-Circ and analyzed for FACS cell cycle analysis. Data are shown as means ± standard error of technical duplicates. N=3.

**C.** Left: cell cycle analysis by flow cytometry (FACS) of RD cells treated with either si-SCR or si-Circ+Lin or si-Lin. N=2. Right: corresponding RNA levels measured by qRT-PCR of circ-ZNF609 (Circ.) and ZNF609 (Lin.). qRT-PCR data are shown as means ± standard error of technical duplicates. The experiment was repeated at least twice.

**D. - E.** Two additional independent western blots in RD (**D**) and RH4 (**E**) cells, in si-SCR and si-Circ conditions (Gapdh hybridization was used as loading control). Corresponding ratio of pRb/Rb protein levels, Akt and p-Akt protein levels (relative to Gapdh) and RNA levels (measured by qRT-PCR of circ-ZNF609 (Circ.), ZNF609 (Lin.) and Rb) are shown. qRT-PCR data are shown as means ± standard error of technical duplicates. The experiment was repeated at least three times (N=3).

**F.** Two additional independent replicates of proteasome inhibition experiment in RD cells treated with either si-SCR or si-Circ: western blot and RNA analysis by qRT-PCR of circ-ZNF609 (Circ.) and ZNF609 (Lin.). qRT-PCR data are shown as means ± standard error of technical duplicates.

**G.** RNA levels measured by qRT-PCR of circZNF609 (Circ.) and ZNF609 (Lin.) in two replicates (#A and #B) of RD and RH4 cells treated with either si-SCR or si-Circ and sent for RNA-Seq analysis. Data are shown as means ± standard error of technical duplicates.

**H.** Hierarchical clustering of samples based on Pearson correlation of gene expression profiles in RD and RH4 cells treated either with si-SCR (si-SCR #A and #B) or with si-Circ (si-Circ #A and #B).

# Supplementary Figures

## 3

### A

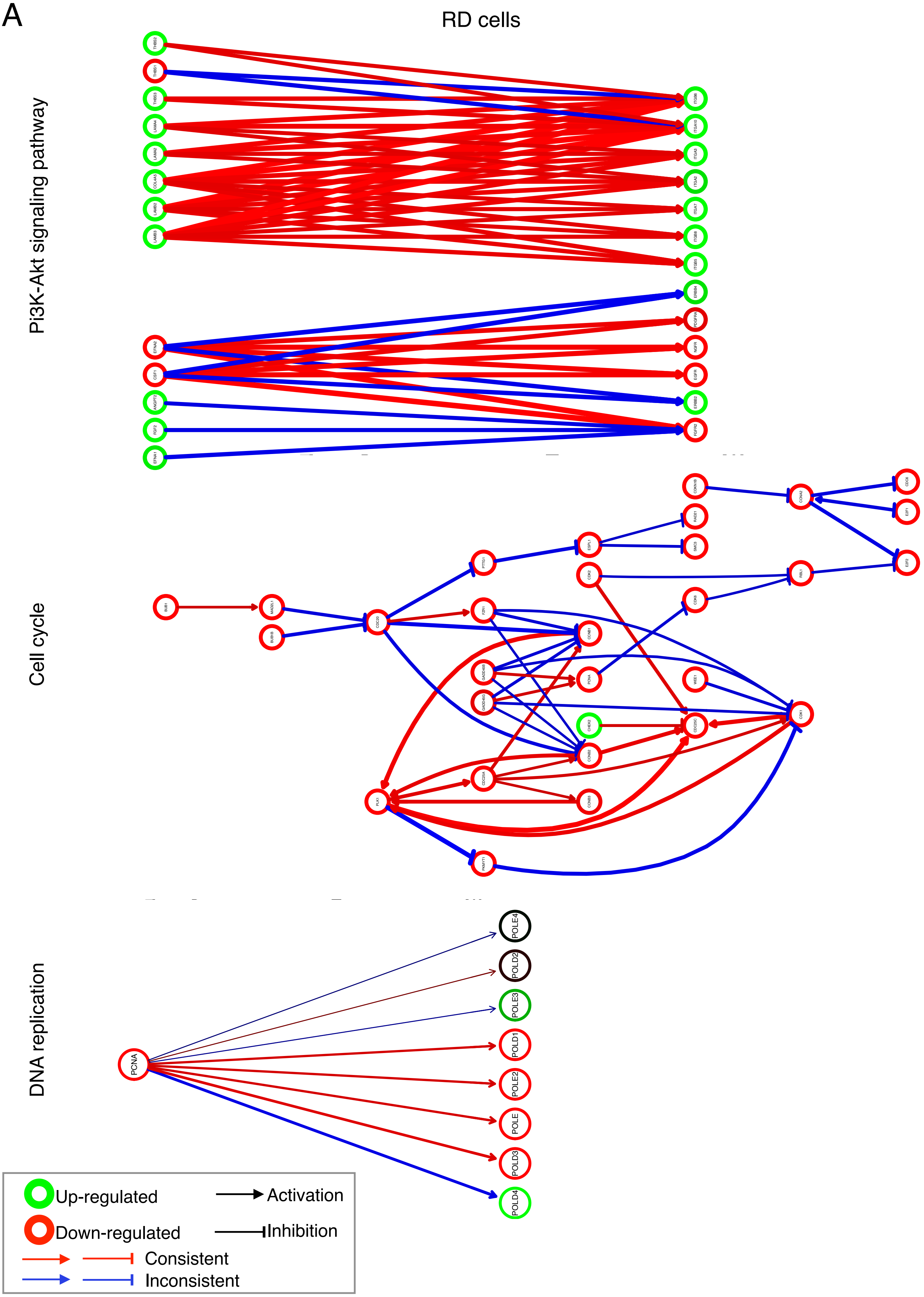

B

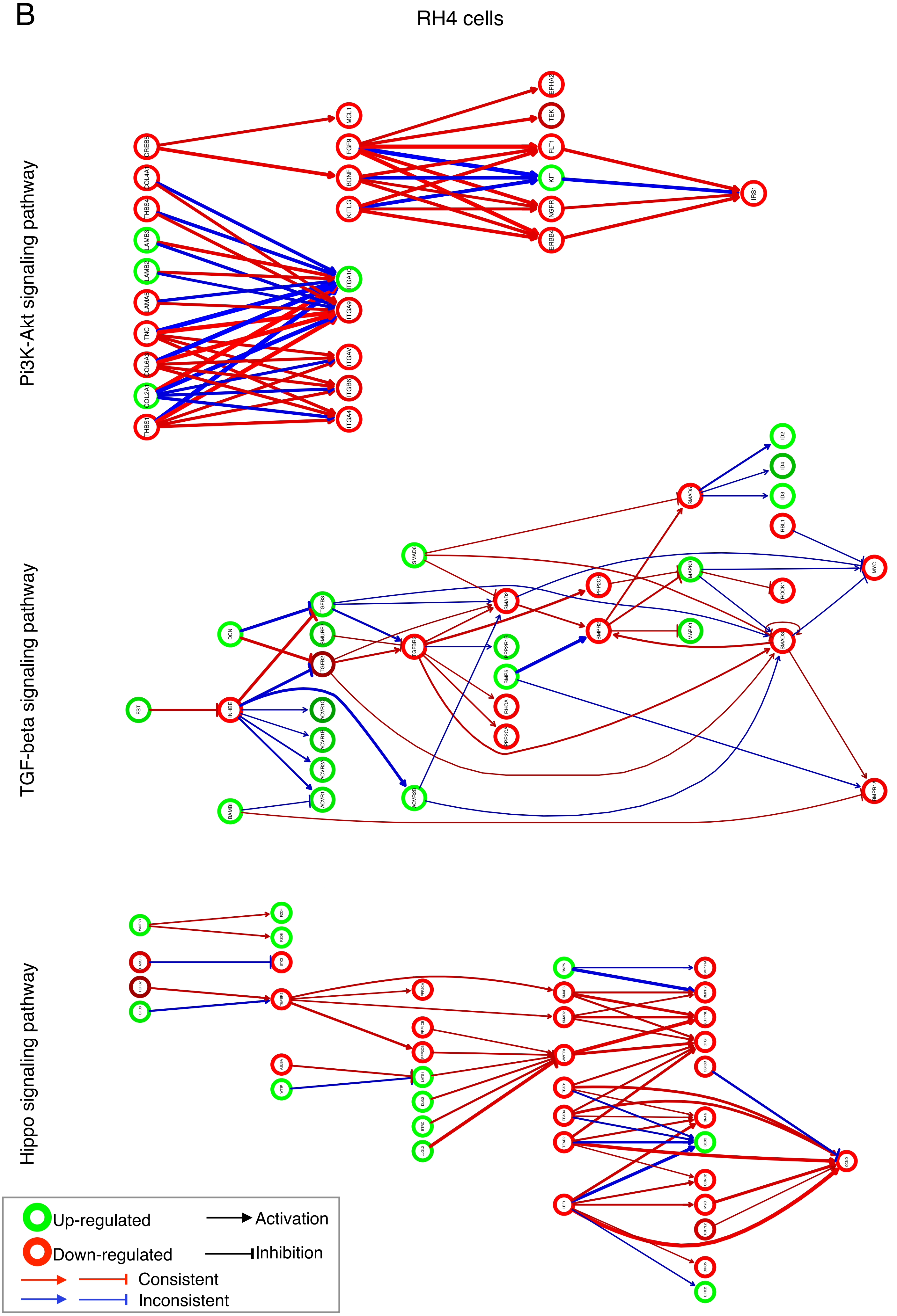

RH4 cells
Pi3K-Akt signaling pathway
CREB5
COL4A1
THBS4
LAMB3
LAMB2
LAMA5
TNC
COL6A3
COL2A1
THBS1
MCL1
FGF9
BDNF
KITLG
ITGA10
ITGA9
ITGAV
ITGB6
ITGA4
EPHA2
TEK
FLT1
KIT
NGFR
ERBB4
IRS1
TGF-beta signaling pathway
FST
INHBE
DCN
BAMBI
TGFB3
SMURF2
TGFB2
ACVR1C
ACVR1B
ACVR2A
ACVR1
SMAD6
ACVR2B
TGFBR2
PPP2R1B
BMP5
RHOA
PPP2CA
SMAD2
PPP2CB
BMPR2
SMAD5
MAPK3
MAPK1
SMAD3
ID2
ID4
ID3
RBL1
ROCK1
MYC
BMPR1A
Hippo signaling pathway
Up-regulated
Down-regulated
Activation
Inhibition
Consistent
Inconsistent

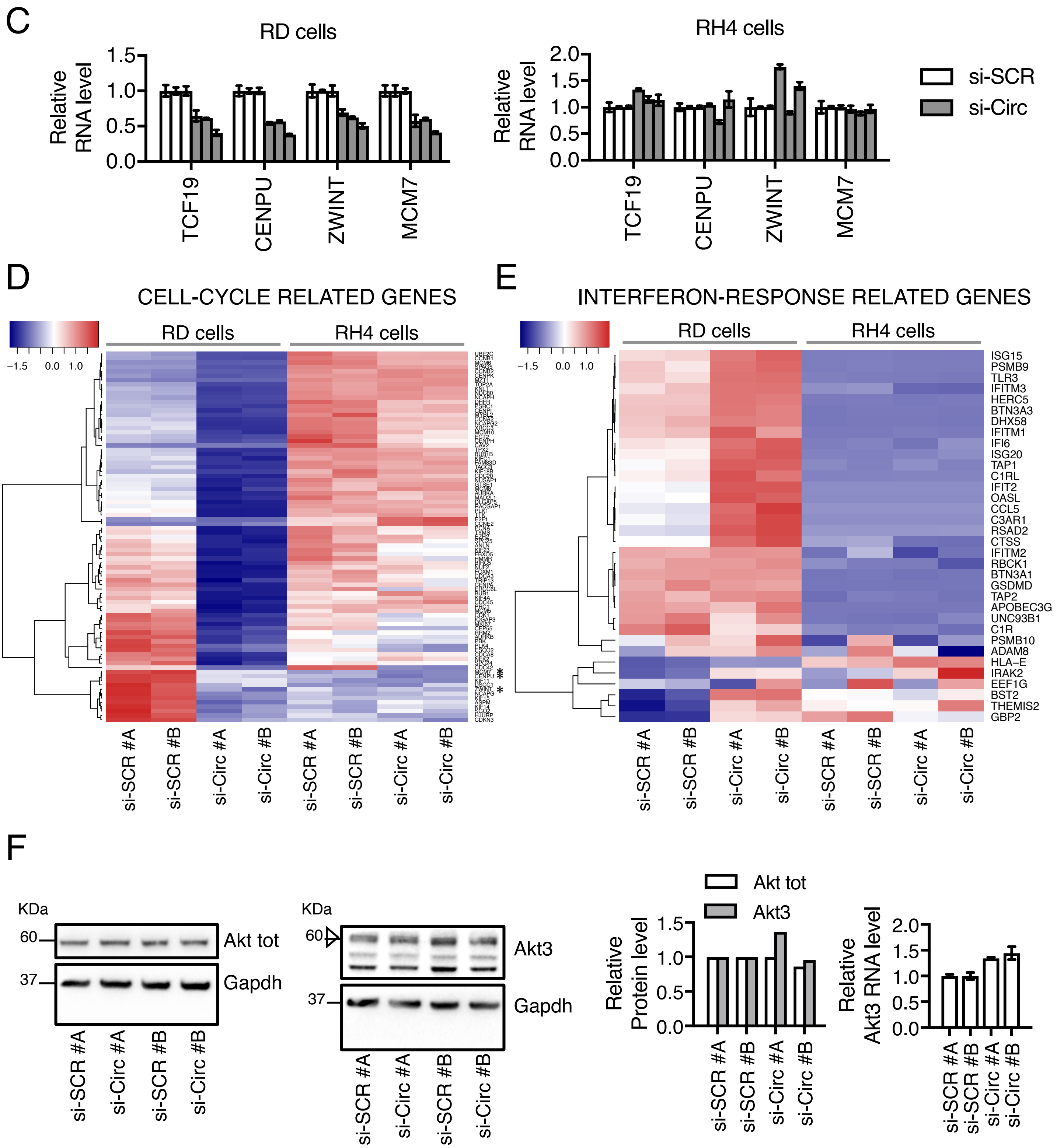


Figure S3

**A.** Some of the most significant GGEA (Gene Graph Enrichment Analysis) results for RD cells (si-SCR vs si-Circ). The clearer the node/edge colour appears, the more significant it is.

**B.** Some of the most significant GGEA (Gene Graph Enrichment Analysis) results for RH4 cells (si-SCR vs si-Circ). The clearer the node/edge colour appears, the more significant it is.

**C.** RNA levels measured by qRT-PCR of some master regulators identified though msVIPER analysis, in RD and RH4 cells, in si-SCR and si-Circ. Data are shown as means ± standard error of technical duplicates. N=3.

**D. - E.** Heatmaps representing differential expression of some cell-cycle related genes (**D**) and some interferon-response related genes (**E**) in RD and RH4 cells in si-SCR and si-Circ, in two replicates (#A and #B) analyzed by RNA-Seq. Expression values are plotted as colour-coded mean-centered log2-transformed RPKM values.

**F.** Western blot analysis of total Akt and Akt3 levels in two independent replicates (#A and #B) of RD cells treated with either si-SCR or si-Circ; corresponding protein levels (relative to Gapdh) and RNA analysis. qRT-PCR data are shown as means ± standard error of technical duplicates. N=2.

Legend of Supplementary Tables

**S1_Differentially expressed genes**
Results of differential expression analyses performed using edgeR software in human primary myoblasts (si-SCR/si-Circ), in RD cells (si-SCR/si-Circ), in RH4 cells (si-SCR/si-Circ) and in RD/RH4 cells.

**S2_Interferon gene expression**
RNA levels of some isoforms of Interferon Alpha (IFN-A) and Beta (IFN-B) expressed in human primary myoblasts were measured by qRT-PCR in a time-course of 6-12-24-48h after either si-SCR or si-Circ transfection. HPRT mRNA was used as a reference target. The table contains Ct (threshold cycle) means of technical replicates. N=2.

**S3_GGEA_rank**
Gene Graph Enrichment Analysis (GGEA) results for RD cells (in si-SCR and si-Circ conditions) and RH4 cells (in si-SCR and si-Circ conditions).

**S4_Oligonucleotides**
List of oligonucleotide pairs (FW and RV) used in this work.

**S5_Read_Number**
Number of reads and mapping statistics for myoblast and RMS samples analyzed by RNA-Seq in this work.

**Supplementary materials and methods**

*RNA-Seq, bioinformatics analysis and statistics*

Total RNA was extracted as described before and treated with DNase I (New England Biolabs, Ipswich, MA, USA) according to the manufacturer's protocol. 2 biological replicates for each condition were performed for RNA-Seq in human primary myoblasts and in RMS samples. Total RNA was sequenced on an Illumina Hiseq 2500 Sequencing system using TruSeq Stranded Total RNA Library Prep Kit with Ribo-Zero treatment at the Institute of Applied Genomics (IGA; Udine, Italy). An average of about 13 million and 30 million 125-nucleotide long paired-end reads were produced for myoblast and RMS samples, respectively. Adapter sequences and poor quality ends were removed using the Trimmomatic software (53); reads with length < 20 nt after trimming were filtered out. Reads were mapped to human GRCh38 genome and Ensembl 90 transcriptome (54) using STAR software (55), with parameters *--outSAMstrandField intronMotif --outFilterType BySJout --outFilterMultimapNmax 20 --alignSJoverhangMin 8 --alignSJDBoverhangMin 1 --outFilterMismatchNmax 999 --outFilterMismatchNoverLmax 0.04 --outFilterIntronMotifs RemoveNoncanonica*l. Number of reads and mapping statistics are reported in Table S5. Htseq-count software (56) was used to count reads mapping to Ensembl 90 human genes; the overlap resolution mode was set to *intersection-strict*. Differential gene expression was performed using edgeR R package (57) after filtering out genes with a CPM (Count Per Million) value less than 1 in at least two samples. edgeR software is particularly suited for experiments with few replicates (58). TMM normalization was applied independently for myoblast and RMS samples. Pearson correlation-based sample hierarchical clusterings, as well as gene expression heatmaps were drawn based on log2-transformed RPKM values, calculated using the edgeR *rpkm* function. Model fitting and testing were performed using *glmFIT* and *glmLRT* functions. FDR cutoff for selecting significant differentially expressed genes was set to 0.05 and absolute log2 fold change was set to > 0.5. Gene Ontology (GO) terms (59) and Reactome pathways (45) enrichment analyses were performed using WebGestalt web server (60) and all genes tested for differential expression as a background; an adjusted p-value cutoff of 0.05 was used to select enriched categories. EnrichmentBrowser R package (61) was employed to perform KEGG pathways (32) based Gene Graph Enrichment analysis (31), using an absolute log2 fold change significance level equal to 0.5 and a statistical significance level equal to 0.05. Transcriptional regulator activity analysis was performed through msviper function included in the Viper R package (33) using RPKMs as expression values; the ARACNe-AP-inferred (62) human sarcoma (63) regulon used in the analysis was taken from the aracne.networks R package, available at https://bioconductor.org/packages/release/data/experiment/html/aracne.networks.html. A p-value cutoff of 0.05 was used to filter results, which were then ranked based on p-value; the top 30 transcriptional regulators were used to draw the msVIPER analysis plot.

*Fluorescent in situ hybridization*

The cells were cultured on pre-coated glass coverslips (0,4 mg/ml Collagen Rat Tail, Corning) and then fixed in 4% paraformaldehyde/PBS (Electron Microscopy Sciences, Hatfield, PA) for 20 min at 4°C. After dehydration steps with ice-cold Ethanol series (50%, 70%, 100%), cells were stored at -20°C in absolute ethanol until use. Detection of circular ZNF609 RNA was performed using Basescope assay (Advanced Cell Diagnostics, Bio-Techne) in accordance with the manufacturer's instructions, with a little modification. Briefly, fixed cells were permeabilized with Protease III (diluted 1:15) before hybridization with BA-HS-ZNF609circRNA-1ZZ-Exon1 probe (ref. 708461), designed by ACD and ordered as a single-plex probe to detect back-splice junction of the circular molecule. A probe specific for exon junction Exon2-Exon3 of ZNF609 mRNA (BA-HS-ZNF609linearRNA-1ZZ- Exon2-3 probe, ref. 798471) and for bacterial RNA dapB, were used as positive and negative controls, respectively. The cells were incubated with probe solution (45ul for each coverslip) at 40°C for 2 hour in the EZ Hybridization oven using the humidity control tray and slide rack (ACD Instruments). Amplification and detection steps were performed using Basescope detection reagents – RED (ref. 322910) and SIGMAFAST™ Fast Red TR/Naphthol AS-MX Tablets (Sigma-Aldrich) for fluorescent signal development. The washings after each amplification step were performed three times for 5 minutes with 2 ml of 1x wash buffer (ADC ref. 310091) at room temperature.

When FISH staining was combined to Immunofluorescence, the samples were washed twice for 5 minutes with 2ml of dd$H_2O$ after SIGMAFAST™ incubation and then blocked with BSA 2%/PBS for 15 minutes at room temperature. eIF3B protein was then detected by using anti-eIF3B/EIF3S9 (#A301-761A, Bethyl, Montgomery, TX, USA) antibody diluted 1:100 in 1% BSA/1% goat serum/PBS overnight at 4 °C. Finally, the immunocomplexes were detected with Alexafluor 488-goat anti-rabbit secondary antibody (ThermoFisher A-11001) diluted 1:200 in 1% BSA/1% goat serum/PBS for 45 minutes at room temperature. After extensive washings with PBS, the cells were incubated with DAPI solution (Sigma, D9542; 1ug/ml/PBS) for 5 minutes at room temperature and then mounted using ProLong Diamond Antifade Mountant (ThermoFischer Scientific, P-36961). Samples were imaged on confocal microscopes (Spinning disk Olympus IX73 and laser scanning *FluoViewFV10i Olympus*) equipped with 60X NA1.35 oil (UPLANSApo) and water (UPLSAP) objectives respectively.

The Z-stacks (200 and 300 nm Z-spacing) were collected with MetaMorph or Fluoview software and merged with maximum intensity projection (MIP) method. All images were processed only in intensity threshold, contrast and brightness. Post-acquisition processing was applied using MetaMorph (Molecular Devices) and FIJI software to the entire image.

Supplementary references


53. Bolger AM, Lohse M, Usadel B. Trimmomatic: A flexible trimmer for Illumina sequence data. Bioinformatics. 2014;30(15):2114–20.
54. Zerbino DR, Achuthan P, Akanni W, Amode MR, Barrell D, Bhai J, et al. Ensembl 2018. Nucleic Acids Res. 2018;46(D1):D754–61.
55. Dobin A, Davis CA, Schlesinger F, Drenkow J, Zaleski C, Jha S, et al. STAR: Ultrafast universal RNA-seq aligner. Bioinformatics. 2013;29(1):15–21.
56. Anders S, Pyl PT, Huber W. HTSeq-A Python framework to work with high-throughput sequencing data. Bioinformatics. 2015;31(2):166–9.
57. Robinson MD, McCarthy DJ, Smyth GK. edgeR: A Bioconductor package for differential expression analysis of digital gene expression data. Bioinformatics. 2009;26(1):139–40.
58. Schurch NJ, Schofield P, Gierliński M, Cole C, Sherstnev A, Singh V, et al. How many biological replicates are needed in an RNA-seq experiment and which differential expression tool should you use? RNA. 2016 Jun;22(6):839–51.
59. Carbon S, Dietze H, Lewis SE, Mungall CJ, Munoz-Torres MC, Basu S, et al. Expansion of the gene ontology knowledgebase and resources: The gene ontology consortium. Nucleic Acids Res. 2017;45(D1):D331–8.
60. Zhang B, Kirov S, Snoddy J. WebGestalt: An integrated system for exploring gene sets in various biological contexts. Nucleic Acids Res. 2005;33(SUPPL. 2):741–8.
61. Geistlinger L, Csaba G, Zimmer R. Bioconductor's EnrichmentBrowser: Seamless navigation through combined results of set- & network-based enrichment analysis. BMC Bioinformatics. 2016;17(1):1–11.
62. Lachmann A, Giorgi FM, Lopez G, Califano A. ARACNe-AP: Gene network reverse engineering through adaptive partitioning inference of mutual information. Bioinformatics. 2016;32(14):2233–5.
63. Weinstein JN, Collisson EA, Mills GB, Shaw KM, Brad A, Ellrott K, et al. The cancer genome atlas pan-cancer analysis project. Nat Genet. 2013;45(10):1113–20
